# Clinical efficacy and prognostic factors of CT-guided radioactive iodine-125 seed implantation for the treatment of superficial soft tissue metastasis: a 12-year retrospective analysis

**DOI:** 10.1186/s13014-024-02475-w

**Published:** 2024-06-24

**Authors:** Weiguang Qiang, Hongbing Shi, Bai Sun, Hao Wang, Chao Wang, Ye Yuan, Wenwei Hu

**Affiliations:** Department of Oncology, The Third Affiliated Hospital of Soochow University, Changzhou, People’s Republic of China

**Keywords:** Radioactive iodine-125 seed implantation, Iodine-125 brachytherapy, Superficial soft tissue metastases, Prognostic factor

## Abstract

**Background:**

Superficial soft tissue metastasis (S-STM) of malignant tumors is uncommon and often brings great pain to patients. However, current treatment options are limited. The purpose of this study was to explore the clinical efficacy and prognostic factors of CT-guided radioactive iodine-125 (^125^I) seed implantation (RISI) for the treatment of S-STM.

**Methods:**

We retrospectively evaluated 132 patients with S-STM who received RISI between June 2010 and July 2022. Local tumor progression-free survival (ltPFS), tumor response, pain control and complication were analyzed. The independent factors affecting ltPFS were screened out using a layered Cox proportional hazards model.

**Results:**

The median follow-up time was 8.3 months (interquartile range [IQR], 4.5–15.3 months). The objective response rate (ORR) was 81.8%. The median ltPFS was 9.1 (95% CI: 6.6, 11.6) months. The Cox proportional hazard regression model revealed that the independent factors influencing ltPFS included KPS score, primary tumor, metastases, boundary, density and postoperative D90 (All *P* < 0.05). After RISI, the rate of pain relief was 92.3%. 66 (84.6%) patients reported pain marked relief, and 6 (7.7%) experienced pain moderate relief. No severe adverse events associated with RISI were observed during follow-up.

**Conclusions:**

CT-guided RISI was associated with high local control and pain relief without severe adverse events and should be considered as a reliable palliative treatment modality for S-STM.

**Trial registration:**

Trial registration Retrospectively registered.

## Background

Superficial soft tissue metastasis (S-STM) from malignant tumors is rare, with a reported incidence of 0.75-9% [1]. Their rapid growth and surrounding invasion can lead to skin rupture or bone destruction, bringing great pain and fear to the patient [2]. Using effective treatment means can obviously relieve the patient’s physical and psychological pain. At present, the traditional treatment methods for S-STM include surgical excision, radiotherapy and systemic chemotherapy. However, S-STM is usually large or ill-defined, resulting in residual tumors that are prone to recurrence or require additional radiotherapy [3]. For inoperable patients, external-beam radiotherapy (EBRT) is an alternative, but the limitation of radiation dose leads to a high probability of recurrence of the lesion, and the associated serious adverse events such as radiation dermatitis, interstitial pneumonia and chronic enteritis limit its use. Although chemotherapy is the main treatment for advanced tumors, it is difficult to maintain high drug concentration in S-STM, resulting in poor therapeutic effects. As a result, the management of these patients remains challenging.

Radioactive iodine-125 (^125^I) seed implantation (RISI) is one of the most promising methods of brachytherapy, which has the advantages of minimally invasive, high local dose, sustained killing of tumors, and relative safety to surrounding normal tissue [4]. It was initially applied to prostate cancer. With the continuous exploration of clinical practice, RISI has been more and more applied in the local treatment and palliative treatment of various malignant tumors, and has achieved satisfactory clinical results [4–6]. However, these organs are located deep in the human body. In the process of puncture, in addition to avoiding important organs and blood vessels, problems related to respiratory movement should be solved, which often lead to complicated treatment plans for RISI. The treatment of S-STM with RISI is simple and safe due to its surface location, but relevant reports are rarely reported. The objective of this study was to evaluate the clinical efficacy and related influencing factors of CT-guided percutaneous brachytherapy with RISI.

## Methods

### Patients

A total of 132 consecutive patients with S-STM treated with CT-guided percutaneous RISI from June 2010 to July 2022 were included in this study. The inclusion criteria: (1) S-STM was confirmed by pathological or radiological diagnosis; (2) Survival time was expected to be ≥ 3 months; (3) pre-operative plan showed that the prescribed doses were satisfactory; (4) The lesions were located in the superficial soft tissue, involving skeletal muscle and/or subcutaneous tissue of the upper and lower limbs, trunk, shoulders, and buttocks.

Exclusion criteria: (1) Primary superficial soft tissue malignant tumors and hematopoietic malignancies; (2) Severe skin or soft tissue infection was present at the site of the lesion; (3) Poor general condition or abnormal coagulation function; (4) Direct extension from tumors originating in bone or adjacent organs. (5) Lack of imaging examinations, such as CT, MRI and PET-CT before or after treatment.

### Instruments

The brachytherapy treatment planning system (TPS, Qilin Co., Ltd., Peking, China) was applied to calculate the ^125^I seed dose distribution based on the American Association of Physicists in Medicine Task Group No. 43 (AAPM TG-43) formalism.

The ^125^I seed (XinKe Pharmaceutical Ltd, Shanghai, China) was shaped as a cylindrical titanium package body with an outer diameter of 0.8 mm, length of 4.5 mm, and wall thickness of 0.05 mm. The gamma rays emitted by ^125^I seeds (5% of 35 keV, 95% of 28 keV) had a half-life of 59.6 days, penetration of 17 mm, and activities of 0.5–0.8 mCi. During the procedure of RISI, 18G implantation needles and an implantation gun (XinKe Pharmaceutical Ltd, Shanghai, China) were used to implant the seeds under CT (Siemens, Germany) guidance.

### CT-guided RISI procedure

**Fig. 1:**
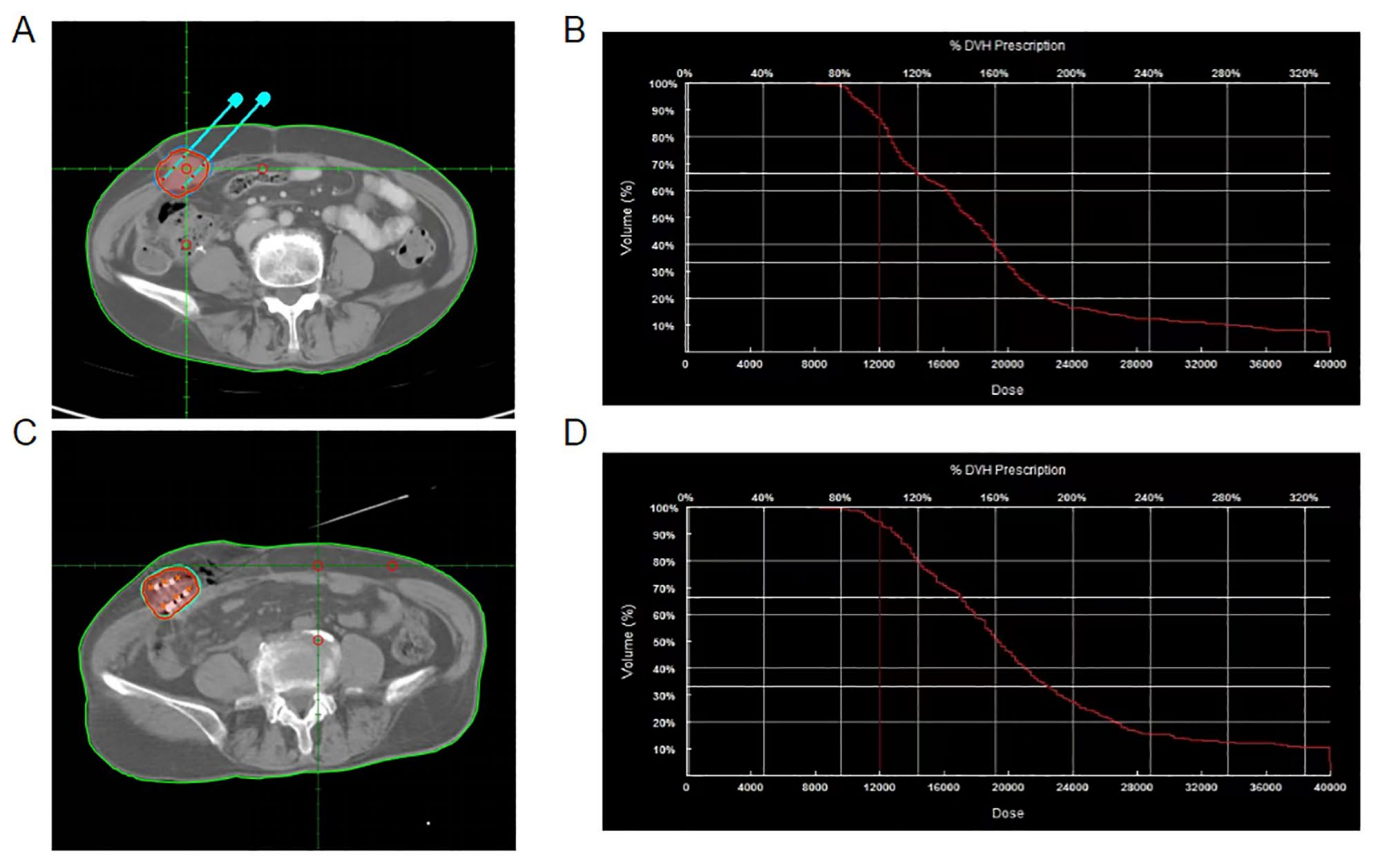
TPS of RISI for pre-plan and postoperative dose verification. (**A**) Preoperative TPS showed the planned puncture path. Red lines represent the tumor’s contour. (**B**) Preoperative dose volume histograms, D90 = 113.7 Gy. (**C**) The distribution of 125I seeds radiation dose after RISI. (**D**) Postoperative dose volume histograms, D90 = 127.4 Gy

All patients underwent preoperative routine examinations, such as blood tests, coagulation function tests and electrocardiograms to rule out any contraindications. Before the procedure, the patients received a CT scan with 5 mm slice thickness. CT image data were transmitted to TPS for pre-plan. The gross tumor volume (GTV) was identified on each transverse image. Then the required amount of ^125^I seeds, activity, and the prescription dose were calculated by TPS. The median prescription dose was 120 Gy (interquartile range [IQR], 100–160 Gy) and the median activity of the ^125^I seed was 0.7 mCi (range, 0.5-0.7mCi). The most recent CT images were imported into TPS for postoperative dose verification. The doses received by 90% of the GTV (D90) were supposed to reach the prescription dose as much as possible, and the doses delivered to the adjacent normal organs were as low as possible (Fig. 1).

The ^125^I seeds were implanted into S-STM under CT guidance, with the patients placed in the appropriate position according to the location of the lesion. The puncture site should avoid the skin that had been invaded by the lesion and the puncture path was established on the CT scan image with reference to preoperative TPS. After local infiltration anesthesia, the 18G seed needle was inserted into the target lesion, and adjustment of the needle position was performed in real-time. Eventually, all of the needles were positioned to the farthest boundary of the tumor while ensuring that the distance between each needle was approximately 1 cm. The ^125^I seeds were implanted into the tumor using a seed implantation gun with a 0.5–1.0 cm distance between two seeds.

A final CT image was performed immediately to ensure uniform spatial distribution of the seeds and to minimize the missed area. Then the CT images were transferred into the TPS for postoperative dose verification. The dosimetric parameters, e.g. D90, were recognized. All procedures followed the recommendations of the International Commission on Radiological Protection.

### Follow-up

The follow-up time was defined as the time interval from initial treatment to death or loss of follow-up. Follow-up of all patients was carried out every 2 months. The tumor diameter, pain condition and physical status of all patients were recorded in detail during follow-up. The primary endpoint was local tumor progression-free survival (ltPFS), which was defined as the duration between the RISI date and the date of local progression or death. Tumor response was evaluated according to the Response Evaluation Criteria in Solid Tumors (RECIST 1.1) [7]: complete response (CR): all target lesions disappear, confirmed at 4 weeks; partial response (PR): baseline lesion total diameter reduction ≥ 30%, confirmed at 4 weeks; stable disease (SD): between PR and PD; progression disease (PD): total length of lesion increased ≥ 20% or new lesions. The objective response rate (ORR) was calculated as (CR + PR) / total number of patients × 100%. Numerical Rating Scale (NRS) score was used to assess the pain degree of the patients before and after treatment. The range of the score was 0–10 points (0 points referred to no pain, 10 points referred to the worst pain). Changes of NRS score in pain versus pre-treatment were categorized as marked improvement (decrease ≥ 50%), moderate improvement (decrease between ≥ 30% and < 50%), stable (between moderate improvement and worse), and worse (increase ≥ 30%) [8]. Treatment-related toxicities were evaluated using the Common Terminology Criteria for Adverse Events (CTCAE) (version 5.0) [9].

### Statistical analysis

Statistical analysis was performed using SPSS 27.0 (IBM, Armonk, NY, USA). Numerical data with normal distribution were expressed as mean value ± standard deviation, whereas data with non-normal distribution were expressed as the median value (IQR). Non-normal distribution data were compared using the Kruskall-Wallis H test. The ltPFS rates were estimated using the Kaplan-Meier method. Univariate and multifactor analyses were conducted using Cox regression. *P* < 0.05 was considered statistically significant.

## Results

### Patient characteristics

A total of 132 patients with S-STM were included in our study, with 46 (34.8%) confirmed by pathology and 86 (65.2%) confirmed by imaging. Their baseline information was shown in Table 1. Of all the patients, 88 (66.7%) were male and 44 (33.3%) were female, with a median age of 65 years (IQR, 56–70 years). 116 (87.9%) patients had KPS scores ≥ 80 and 78 (59.1%) patients had local pain with a median NRS pain score of 4.0 (IQR, 2.8-6.0). Of the 25 lung cancer patients, 23 (92%) had non-small cell lung cancer (NSCLC), and 2 (8%) had poorly differentiated cancer. Additionally, 97 (73.5%) had previously received systemic chemotherapy and 10 (7.6%) had undergone local radiotherapy. Moreover, the median maximum diameter of the tumor was 4.2 cm (IQR, 2.6–6.0 cm), and 47 (35.6%) patients had S-STM without any other organ metastases. The median postoperative D90 was 123 Gy (IQR, 104–145 Gy).

**Table 1:** Clinical characteristics of 132 patients

Characteristics	Values
Gender	
Male Female	88(66.7%) 44(33.3%)
Age (years, median, IQR)	65 (56–70)
KPS	
≥ 80 < 80	116 (87.9%) 16 (12.1)
Primary tumor	
Colorectal carcinoma Lung carcinoma Head-neck carcinoma Gastric carcinoma Skin and soft tissue carcinoma Urinary system carcinoma Hepatobiliary carcinoma Breast carcinoma Reproductive system carcinoma Thymic carcinoma Esophageal carcinoma Unknown	36 (27.3%) 25 (18.9%) 15 (11.4%) 11 (8.3%) 10 (7.6%) 7 (5.3%) 7 (5.3%) 6 (4.5%) 5 (3.8%) 5 (3.8%) 4 (3.0%) 1 (0.8%)
Pathological grade	
High-grade Low-grade Unknown	61 (46.2%) 44 (33.3%) 27 (20.5%)
Number of metastases	
1 ≥ 2	47 (35.6%) 85 (64.4%)
Seed implantation site	
Thoracic wall Abdominal wall Head and neck Arms and legs Other ^a^	48(36.3%) 47(35.6%) 17(12.9%) 5(3.8%) 15(11.4%)
Maximum diameter (cm, median, IQR)	4.2(2.6-6.0)
Previous radiotherapy	
Yes No	10(7.6%) 122(92.4%)
Previous chemotherapy	
Yes No	97(73.5%) 35(26.5%)
Postoperative systemic therapy ^b^	
Yes No	97(73.5%) 35(26.5%)
cancer pain (NRS score)	
0 1–3 4–6 7–10	54(40.9%) 34(25.8%) 41(31.1%) 3(2.3%)

^a^ Armpit and groin. ^b^ Including chemotherapy, targeted therapy, immunotherapy or combination therapy

### Tumor response and pain control

**Fig. 2:**
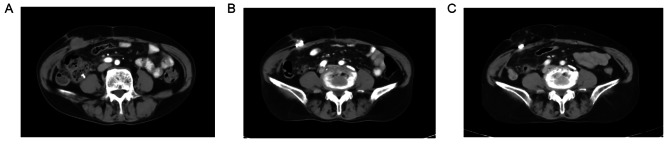
Images from a 68-year-old patient with S-STM of colorectal cancer. (**A**) Preoperative CT enhanced scanning images showed an abdominal wall lesion. (**B**) Two months after RISI, the lesions have shrunk significantly. (**C**) Six months after RISI, CR was achieved on the image and 125I seeds aggregation was left

The 132 patients were followed up for a median duration of 8.3 months (IQR, 4.5–15.3 months). According to RECIST criteria and the follow-up imaging data, all patients were evaluated for local tumor response after RISI. As shown in Fig. 2; Table 2, there were 32 (24.2%) cases of CR, 76 (57.6%) cases of PR, 15 (11.4%) cases of SD, and 9 (6.8%) cases of PD. The objective response rate (ORR) was 81.8%.

**Table 2:** Local control and pain improvement after RISI

	*N* of patients
Local control	
CR	32 (24.2%)
PR	76 (57.6%)
SD	15 (11.4%)
PD	9 (6.8%)
ORR	108 (81.8%)
Pain improvement	
Marked (NRS decrease ≥ 50%)	66 (84.6%)
Moderate (NRS decrease > 30%)	6 (7.7%)
Stable	4 (5.1%)
Worse (NRS increase > 30%)	2 (2.6%)

**Fig. 3:**
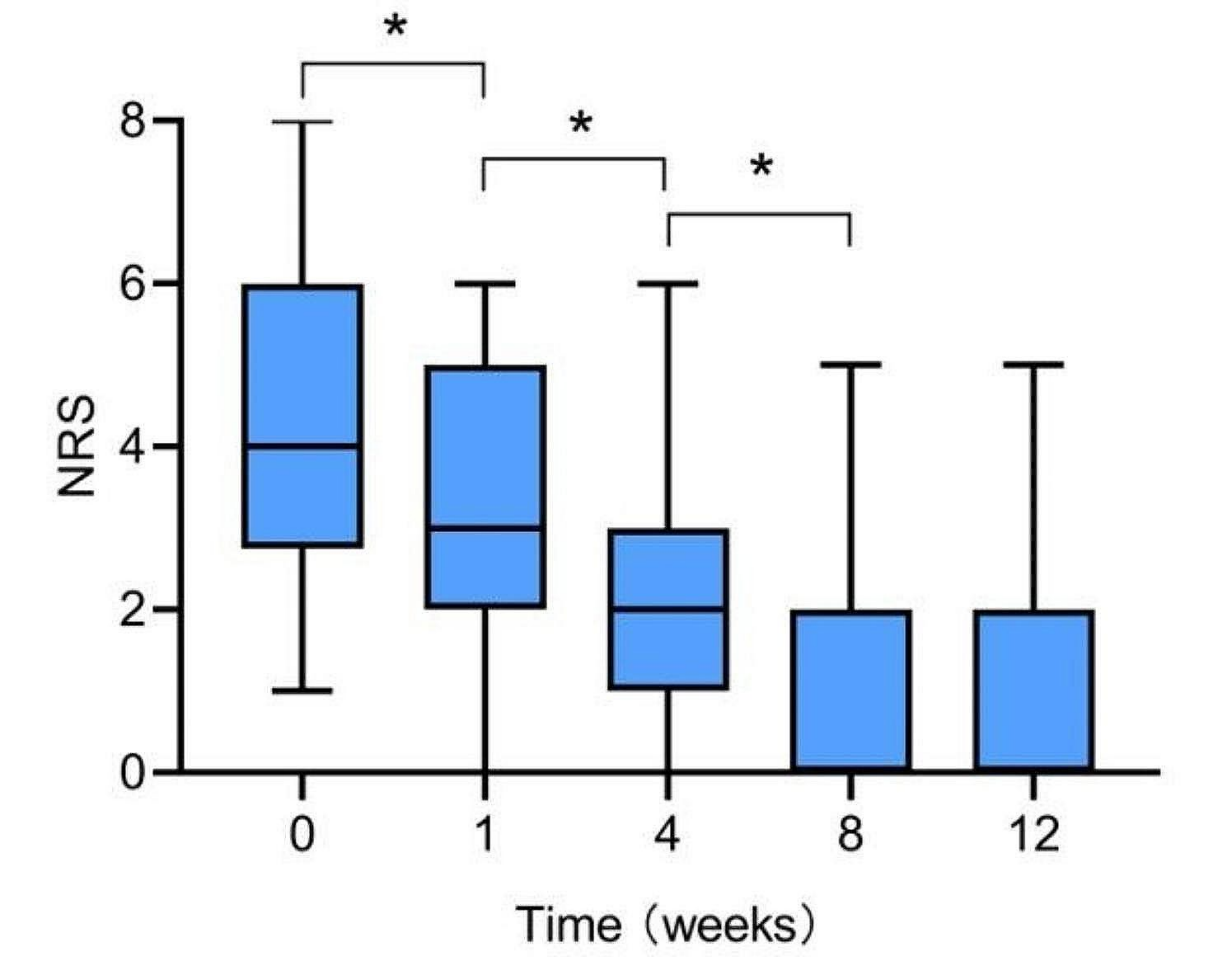
NRS score was tested before RISI and 1 week, 1 month, and 3 months after RISI and showed that the difference in NRS scores between adjacent time points was statistically significant (* *p* < 0.05), except for 12 weeks and 8 weeks (*P* = 0.815). All data are expressed as median value (IQR)

The pain improvement of patients is shown in Table 2. After RISI, 92.3% of patients showed an improvement in pain. Of these, 66 (84.6%) patients reported pain marked relief, and 6 (7.7%) experienced pain moderate relief. In addition, compared to before brachytherapy, NRS scores decreased significantly at 1, 4 and 8 weeks post-treatment (all *p* < 0.05). But the NRS scores at 8 weeks and 12 weeks were not statistically significant (*p* > 0.05) (Fig. 3).

### ltPFS and affecting factors

The median ltPFS was 9.1 (95% CI: 6.6, 11.6) months. In the Cox proportional hazard regression model, as shown in Table 3, the univariable analysis identified KPS, primary tumor, pathological grade, number of metastases, D90, maximum diameter, boundary, shape, density and necrosis to be related to ltPFS (All *P* < 0.05). Then these factors were included in Cox multivariate analysis. The results showed that the independent factors influencing ltPFS included KPS, primary tumor, number of metastases, boundary, density and D90 (All *P* < 0.05, Table 3).

**Table 3:** Predictive factors of ltPFS in patients with S-STM who underwent RISI

Characteristics	Univariable Analysis		Multivariable Analysis	
	HR (95% CI)	*P* value	HR (95% CI)	*P* value
Gender				
Male	1.0			
Female	0.927(0.631–1.364)	0.702		
Age (years)				
< 60	1.0			
≥ 60	1.302(0.885–1.914)	0.180		
KPS				
≥ 80	1.0		1.0	
< 80	**4.110(2.320–7.286)**	**< 0.001**	**2.391(1.302–4.394)**	**0.005**
Primary tumor				
Colorectal carcinoma	1.0		1.0	
Lung carcinoma	1.510(0.887–2.571)	0.129	**2.433(1.365–4.335)**	**0.003**
Head-neck carcinoma	**0.498(0.253–0.981)**	**0.044**	1.409(0.657–3.022)	0.378
Gastric carcinoma	0.915(0.451–1.857)	0.806	1.781(0.840–3.775)	0.132
Skin and soft tissue carcinoma	**2.442(1.157–5.156)**	**0.019**	**2.721(1.172–6.317)**	**0.020**
Others ^a^	0.665(0.398–1.111)	0.119	1.327(0.745–2.364)	0.337
Pathological grade				
High-grade	1.0		1.0	
Low-grade	**1.544(1.025–2.325)**	**0.037**	1.248(0.774–2.011)	0.364
Unknown	1.154(0.705–1.890)	0.569	0.808(0.470–1.389)	0.441
Number of metastases				
1	1.0		1.0	
≥ 2	**2.030(1.364–3.021)**	**< 0.001**	**1.980(1.296–3.027)**	**0.002**
Seed implantation site				
Thoracic wall	1.0			
Abdominal wall	1.339(0.472–3.802)	0.584		
Head and neck	1.064(0.567–1.999)	0.846		
Arms and legs	1.351(0.720–2.536)	0.349		
Others ^b^	0.833(0.389–1.784)	0.639		
D90 (Gy)				
< 120	1.0		1.0	
≥ 120	**0.306(0.208–0.451)**	**< 0.001**	**0.371(0.235–0.586)**	**< 0.001**
Distance from skin (cm)^c^				
< 1	1.0			
≥ 1	0.851(0.586–1.237)	0.398		
Maximum diameter (cm)				
≤ 3	1.0		1.0	
3–5	**1.72(1.083-2.745-)**	**0.022**	0.800(0.429–1.493)	0.483
≥ 5	**4.202(2.606–6.776)**	**< 0.001**	1.381(0.635–3.008)	0.416
Clear boundary				
No	1.0		1.0	
Yes	**0.258(0.170–0.390)**	**< 0.001**	**0.501(0.297–0.846)**	**0.010**
Regular shape				
No	1.0		1.0	
Yes	**0.357(0.239–0.533)**	**< 0.001**	1.126(0.610–2.079)	0.704
Uniform density				
No	1.0		1.0	
Yes	**0.309(0.205–0.464)**	**< 0.001**	**0.546(0.337–0.886)**	**0.014**
Necrosis				
No	1.0		1.0	
Yes	**2.927(1.823–4.701)**	**< 0.001**	1.049(0.523–2.104)	0.893
Previous radiotherapy				
No	1.0			
Yes	1.491(0.777–2.860)	0.230		
Previous chemotherapy				
No	1.0			
Yes	1.321(0.865–2.017)	0.198		
Postoperative systemic therapy				
No	1.0			
Yes	1.056(0.688–1.621)	0.802		

^a^ Including urinary system carcinoma, hepatobiliary carcinoma, breast carcinoma, reproductive system carcinoma, thymic carcinoma, esophageal carcinoma, unknown. ^b^ Armpit and groin. ^c^ The shortest distance of S-STM from the skin

**Fig. 4:**
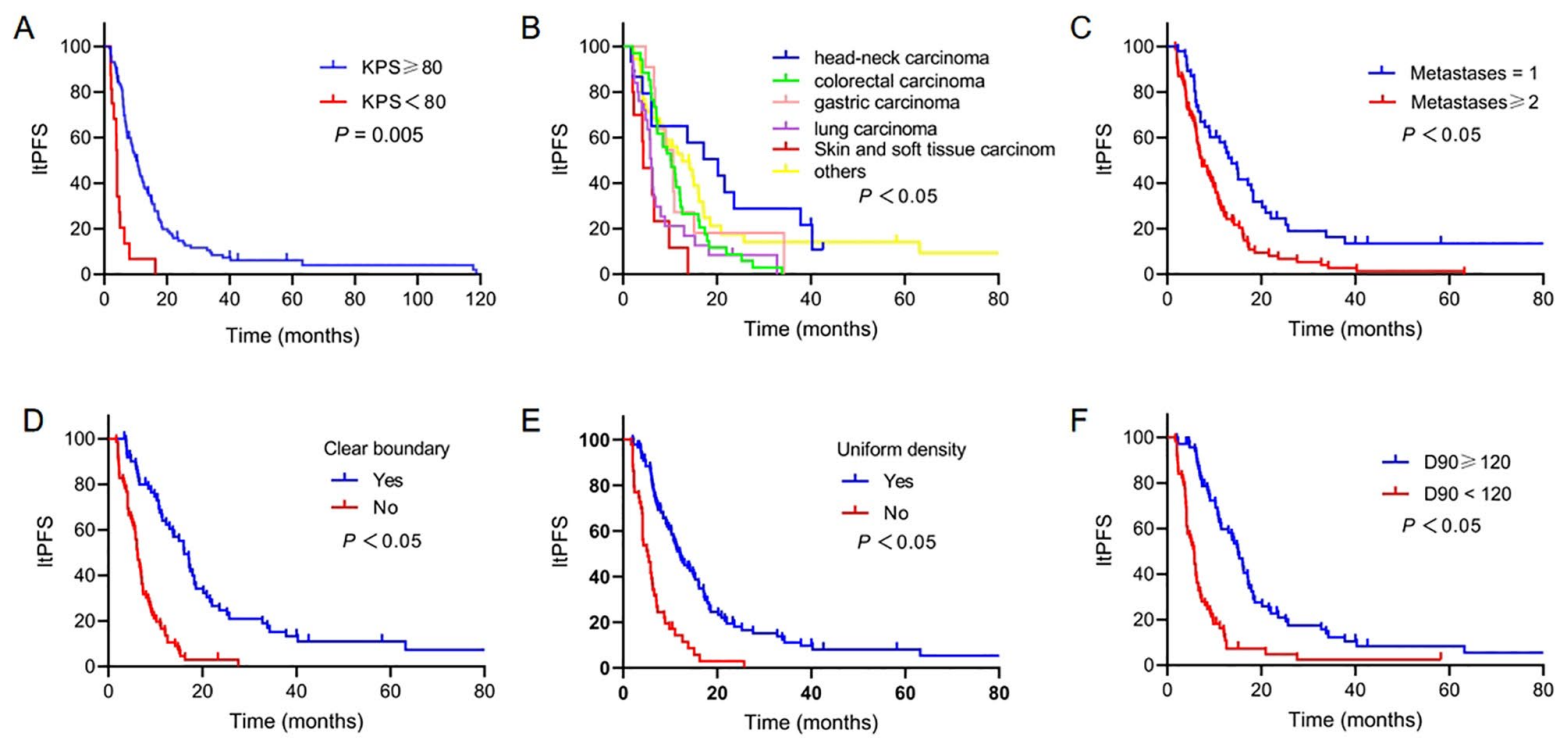
Kaplan-Meier curves showing ltPFS. (**A**) KPS scores. (**B**) Primary tumors. (**C**) Number of metastases. (**D**) Boundary. (**E**) Density. (**F**) D90

Multivariate analysis suggested that the ltPFS of patients with D90 ≥ 120 Gy was significantly better than that of patients with D90 < 120 Gy (HR 0.371; 95% CI 0.235, 0.586; *P* = 0.001). Besides, patients who had clear boundary (HR 0.501; 95% CI 0.297, 0.846; *P* = 0.01) and uniform density (HR 0.546; 95% CI 0.337, 0.886; *P* = 0.01) on scans had a longer ltPFS. While lower ltPFS was observed in patients with other metastases in addition to S-STM (HR 1.980; 95% CI 1.296, 3.027; *P* = 0.002). The poor performance indicated a poor prognosis. Patients with KPS < 80 had a lower ltPFS (HR 2.391; 95% CI 1.302, 4.394; *P* = 0.005) than patients with KPS ≥ 80. Additionally, the primary tumor was also an independent factor affecting ltPFS (*P* = 0.025). Lung carcinoma (HR 2.433; 95% CI 1.365, 4.335; *P* = 0.003) and skin and soft tissue carcinoma (HR 2.721; 95% CI 1.172, 6.317; *P* = 0.02) were associated with worse ltPFS than other tumors (Fig. 4).

### Complications

Adverse events are shown in Table 4. No severe adverse events with Grade 3 or 4 associated with ^125^I brachytherapy were observed during follow-up. Puncture point pain was recorded in 14 (10.6%) patients, postoperative fever was recorded in 5 (3.8%) patients, local bleeding was recorded in 19 (14.4%) patients, and radiation dermatitis was recorded in 3 (2.3%) patients. Except for one patient with Grade 2 radiation dermatitis, all the other adverse events were Grade 1. These adverse events were controlled with symptomatic therapy or subsided spontaneously within a short time. After RISI, there were no adverse events of skin rupture or infection.

**Table 4:** Adverse events after RISI

Adverse events	Any grade	Grade 1	Grade 2	Grade 3	Grade 4
Puncture point pain	14(10.6%)	14(10.6%)	0	0	0
Fever	5(3.8%)	5(3.8%)	0	0	0
Infection	0	0	0	0	0
Local bleeding	19(14.4%)	19(14.4%)	0	0	0
Dermatitis radiation	3(2.3%)	2(1.5%)	1(0.8%)	0	0

## Discussion

S-STM represents a relatively rare form of metastasis, mostly originating from cancers of the lung, gastrointestinal tract, head and neck and breast, which is associated with dismal prognosis and indicates high underlying tumor burden [10]. Several studies estimated the mean survival time of patients was 7.5-9 months once S-STM was diagnosed [11, 12]. Recently, El Abiad et al. [13] conducted a retrospective review of 1341 patients with primary esophageal cancer and found that 25 (1.9%) patients had S-STM. Ten patients received local treatment to the metastatic focus, the median survival times of whom were 11.1 months and 4 months for those who did not (*P* = 0.020). This is an indication that local intervention is associated with improved prognosis for selected patients. In addition, S-STM is typically not considered unless the patient develops a palpable mass, which can ulcerate, bleed, and be very painful. Uncontrolled S-STM adversely affects body image and quality of life in the end. Therefore, local treatment is indicated for patients with S-STM, especially if they are symptomatic.

Unfortunately, S-STM is often difficult to manage clinically as there are simply too few cases to make any conclusion. Surgical excision of a small and isolated metastasis is the treatment of choice, which offers optimal local control and prolongs survival. However, only a small portion of the patients have the chance of surgical excision. Moreover, resection of such tumors may lead to difficulty in wound healing, and even require skin grafting treatment [14]. EBRT is a valid option if the area has not already been treated with EBRT, but it can cause relevant complications such as skin changes (erythema, ulceration and fibrosis) and muscle contractures [15]. Although systemic therapies is the main treatment for advanced tumors, the primary tumor shrunk but the S-STM did not after the patients received systemic therapies. Electrochemotherapy (ECT) is a local treatment of solid tumors by combining permeabilizing electric pulses and non-permeating anticancer drugs with high intrinsic cytotoxicity, facilitating drugs delivery into the cells. Retrospective and prospective studies have shown that ORR achieved 83.2-92.3% after ECT treatment for superficial tumors. However, in a large percentage (50%) of patients, relatively fast locoregional progression was observed, which was managed with additional ECT cycles. Furthermore, to achieve a high response rate, the tumor size needs to be smaller than 2 cm. It is also worth noting that local dermatological toxicity is also very serious [16–18]. In 2023, Martina et al. [19] conducted a systematic review of the combination of ECT and EBRT for tumor treatment. This review suggested that ECT plus EBRT demonstrated superior tumor response compared to that under single therapies. However, prior to introducing a combination of two local treatments for cancer into clinical practice, careful consideration must be given to the risk of overlapping toxicity. Unfortunately, all clinical studies lacked indicators of treatment effectiveness, such as ORR, and provided limited toxicity data.

A series of clinical studies have demonstrated that RISI, as permanent interstitial brachytherapy, is widely used in various solid tumors and has achieved significant clinical effects [20–22]. Compared with traditional EBRT, ^125^I seeds can confine the high-dose area to the tumor, enhancing the antitumor effect with a limited influence on surrounding normal tissues. In addition, by providing continuous low-dose irradiation, ^125^I seeds can keep cell cycle arrest and promote tumor stem cell apoptosis, which may improve efficacy [23]. However, in the actual operation process, the protection of adjacent important organs, the obstruction of bones and the movement of the organs can affect the accuracy of implantation, leading to the inability of implantation to accurately fulfill the preoperative plan and greatly reducing the therapeutic effect. RISI is theoretically well suited for the treatment of S-STM without aforementioned problems because the lesion is relatively superficial and fixed.

In 1993, Mittal et al. [24] reported three patients with chest wall metastases, who were treated with CT-guided RISI. In all patients, significant improvement of symptoms and tumor shrinkage were observed, which was the first indication that RISI may be applied to effectively treat S-STM. Afterwards, Jiang et al. [14] conducted a retrospective analysis of the efficacy and safety of RISI for refractory chest wall metastasis or recurrence under CT guidance. Among all the 20 patients, ORR was 75% (15% CR, 60% PR). In another retrospective study [25], 21 patients with 28 abdominal wall metastases who received RISI under US guidance were retrospectively reviewed. ORR was 78.6%, 64.3% and 52.4% after 3, 6, and 12 months. It is noteworthy that in this study, 7 cases of US-guided seed implantation required supplemental procedures due to incomplete tumor coverage, seed displacement, or uneven distribution. Recently, Jiang et al. [26] published a study that included 19 patients with 22 recurrent chest wall cancer treated with 3D-printing non-coplanar template-assisted CT-guided RISI. CR was observed in 4/22 (18.1%), PR in 13/22 (59.1%) of the cancers and ORR was 77.2%. Until now, our report was the only published study with over 100 cases and the results were similar to these previous studies. CR was observed in 32 (24.2%) and PR in 76 (57.6%) of cases. ORR was 81.8%, demonstrating the efficacy of RISI in local tumor control of S-STM.

Most of the previous reports have only observed the tumor response rate of S-STM treated with RISI, and few studies have examined ltPFS and its prognostic factors. In this study, the median ltPFS was 9.1 (95% CI: 6.6, 11.6) months. Independent prognostic factors for ltPFS were identified as KPS scores, number of metastases, primary tumor, density, boundary, and D90, using the Cox proportional hazards model. Based on clinical experience, KPS score < 80 and multiple metastases often suggest a relatively short survival time, leading to a lower ltPFS. Jiang et al. [27] studied 113 patients who underwent RISI after EBRT or surgery of recurrent head and neck squamous carcinoma and found that KPS score was significantly associated with the local control rate and OS of patients. Parry et al. [28] found that any form of treatment can significantly improve survival time in patients with oligometastases. Moreover, the primary tumor was also an independent factor affecting ltPFS. Lung carcinoma and skin and soft tissue carcinoma were associated with worse ltPFS than other tumors. Pretell-Mazzini et al. [29] summarized previous reports on S-STM and examined the relationships between prognosis and the type of primary tumor and they concluded that lung cancer had a poor prognosis. D90 was related to local control, which was consistent with the findings of Ji et al. and Jiang et al. [22, 27]. This study showed that ltPFS was longer with D90 ≥ 120 Gy than with D90 < 120 Gy, which was in line with clinical experience. Radiologically, the density and boundary of the lesions reflect the malignant degree and invasiveness of tumors. The uneven density and unclear boundaries also represent a poor prognosis. Moreover, these imaging features not only affect the accuracy of the preoperative TPS, but also affect rational placement of the intraoperative ^125^I seeds. These limitations can result in incomplete target coverage, thereby making it difficult to obtain long ltPFS. Previous studies [4, 30] have shown that the maximum diameter is an independent factor affecting the ltPFS of RISI. The same conclusion was not obtained in this study. The observed difference may result from a wider variety of primary cancers and different anatomical locations of the lesions.

S-STM of malignant tumors has been widely reported to be accompanied by substantial pain that lowers the quality of life. Tsuchie et al. [2] found that 75% of patients with S-STM had pain, which was similar to the result of this study. Thus, the aims of treating S-STM were the palliation of symptoms, reducing the complications caused by local tumor invasion, and improving quality of life. In this study, the pain relief rate was 92.3%, with 84.6% of patients experiencing significant relief, which is remarkably high for those who have been suffering from chronic pain. Meanwhile, the incidence of complications associated with ^125^I brachytherapy is consistent with previous reports [6]. No severe adverse events were observed after RISI. The majorities of complications were manageable with symptomatic therapy or subsided spontaneously within a short time. Skin is the organ at risk in the treatment of S-STM with RISI, and many lesions in this study are adjacent to the skin. Surprisingly, only one patient developed Grade 2 radiation dermatitis, which was due to significant tumor retreat within a short period of time, resulting in a redistribution of radiation dose. Therefore, CT-guided RISI may be a feasible and safe treatment for patients with S-STM.

This study was limited by its single-arm and single-center retrospective nature. In addition, it is difficult to conduct a controlled study to compare with other local treatments due to the large number of primary tumor types. However, the study was the first study with over 100 cases investigating Clinical efficacy and prognostic factors of RISI for S-STM obtaining good results. Therefore, we will consider a prospective controlled study in the future.

## Conclusions

S-STM often presents as a mass and persistent pain, seriously affecting the quality of life. Besides, the lesion is located in a superficial location, around where there are no important tissues and organs, making it the most suitable for RISI treatment. This study also confirmed that RISI is an effective and safe therapy in patients with S-STM. Moreover, KPS ≥ 80, most malignant tumors excluding lung, skin and soft tissue carcinoma, oligometastasis, clear boundary, uniform density and high D90 were associated with better local control. The sample size of this study was relatively large, thus providing an important reference for making clinical decisions and conducting future research.

## Data Availability

The data presented in this study are available on request from the corresponding author in an anonymized form after data privacy check. The data are not publicly available due to data privacy regulations.

## Abbreviations

S-STM: Superficial soft tissue metastasis
^125^I: Iodine-125
RISI: Radioactive iodine-125 seed implantation
ltPFS: Local tumor progression-free survival
IQR: Interquartile range
ORR: The objective response rate
EBRT: External-beam radiotherapy
TPS: Treatment planning system
GTV: The gross tumor volume
CR: Complete response
PR: Partial response
SD: Stable disease
PD: Progression disease
NRS: Numerical Rating Scale
ECT: Electrochemotherapy
